# Supplementary material for: Association between oral microbiome and seven types of cancers in East Asian population: a two-sample Mendelian randomization analysis
Source: Front Mol Biosci. 2023 Nov 21;10:1327893. doi: 10.3389/fmolb.2023.1327893 (PMC10702768; doi:10.3389/fmolb.2023.1327893)
Supplement: Supplementary file 1 [file DataSheet1.ZIP › Supplementary Materials/MR plots of saliva/saliva═╝/Breast cancer/pheno.100_to_breast cancer_forest.pdf]

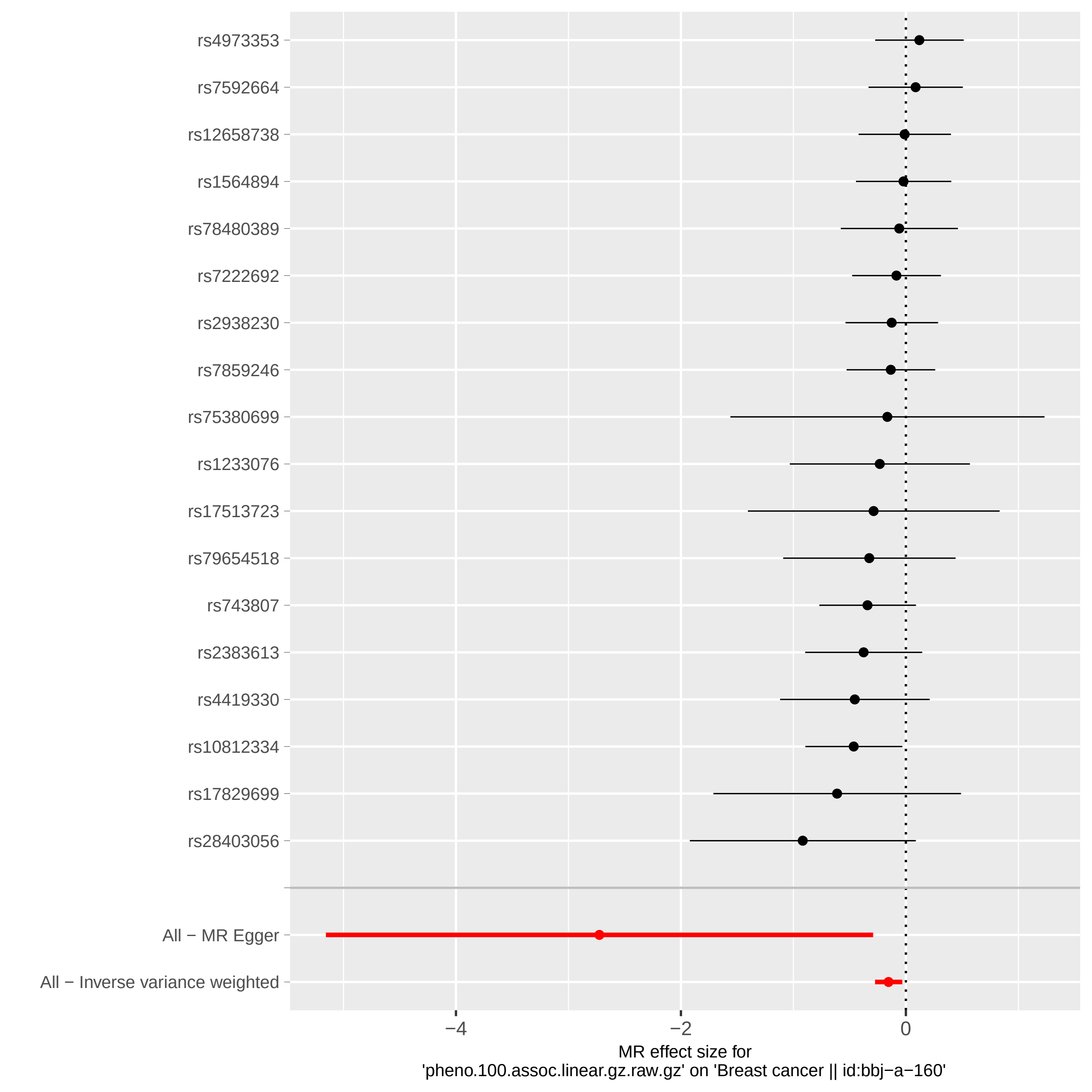
rs4973353
rs7592664
rs12658738
rs1564894
rs78480389
rs7222692
rs2938230
rs7859246
rs75380699
rs1233076
rs17513723
rs79654518
rs743807
rs2383613
rs4419330
rs10812334
rs17829699
rs28403056
All − MR Egger
All − Inverse variance weighted
−4
−2
0
MR effect size for
'pheno.100.assoc.linear.gz.raw.gz' on 'Breast cancer || id:bbj−a−160'
